# Multi-Spectroscopic Analysis of Seed Quality and ^13^C-Stable-Iotopologue Monitoring in Initial Growth Metabolism of *Jatropha curcas* L.

**DOI:** 10.3390/metabo4041018

**Published:** 2014-11-13

**Authors:** Takanori Komatsu, Risa Ohishi, Amiu Shino, Kinya Akashi, Jun Kikuchi

**Affiliations:** 1 RIKEN Center for Sustainable Resource Science, 1-7-22 Suehirocho, Tsurumi-ku, Yokohama, Kanagawa 230-0045, Japan; E-Mails: takanori.komatsu@riken.jp (T.K.); amiu.shino@riken.jp (A.S); 2 Graduate School of Medical Life Science, Yokohama City University, 1-7-29 Suehirocho, Tsurumi-ku, Yokohama, Kanagawa 230-0045, Japan; E-Mail: b.stone23@tsurumi.yokohama-cu.ac.jp; 3 Faculty of Agriculture, Tottori University, 4-101 Koyama-cho, Tottori 680-8553, Japan; E-Mail: akashi.kinya@muses.tottori-u.ac.jp; 4 RIKEN Biomass Engineering Program Cooperation Division, RIKEN Center for Sustainable Resource Science, 1-7-22 Suehirocho, Tsurumi-ku, Yokohama, Kanagawa 230-0045, Japan; 5 Graduate School of Bioagricultural Sciences, Nagoya University, 1 Furo-cho, Chikusa-ku, Nagoya, Aichi 464-0810, Japan

**Keywords:** NMR, stable-isotope labeling, quality examination, isotopic analysis

## Abstract

In the present study, we applied nuclear magnetic resonance (NMR), as well as near-infrared (NIR) spectroscopy, to *Jatropha curcas* to fulfill two objectives: (1) to qualitatively examine the seeds stored at different conditions, and (2) to monitor the metabolism of *J. curcas* during its initial growth stage under stable-isotope-labeling condition (until 15 days after seeding). NIR spectra could non-invasively distinguish differences in storage conditions. NMR metabolic analysis of water-soluble metabolites identified sucrose and raffinose family oligosaccharides as positive markers and gluconic acid as a negative marker of seed germination. Isotopic labeling patteren of metabolites in germinated seedlings cultured in agar-plate containg ^13^C-glucose and ^15^N-nitrate was analyzed by zero-quantum-filtered-total correlation spectroscopy (ZQF-TOCSY) and ^13^C-detected ^1^H-^13^C heteronuclear correlation spectroscopy (HETCOR). ^13^C-detected HETOCR with ^13^C-optimized cryogenic probe provided high-resolution ^13^C-NMR spectra of each metabolite in molecular crowd. The ^13^C-^13^C/^12^C bondmer estimated from ^1^H-^13^C HETCOR spectra indicated that glutamine and arginine were the major organic compounds for nitrogen and carbon transfer from roots to leaves.

## 1. Introduction

Jatropha (*Jatropha curcas* L.) is a drought-resistant shrub that originated from Central America and is considered a prospective economically relevant plant due to the high oil seed content [1,2]. Its seed contains 30%–45% oil, with a high amount triglycerides consisting of, mainly, oleic and linoleic acid, as well as toxic compounds, such as phorbol ester, lectin dimers, and curcin [3]. *J. curcas* is considered a semi-wild plant and has not been fully domesticated [4], although its whole genome has been sequenced and reported in 2011 [5,6]. Therefore, its oil productivity is variable, making it difficult to predict yields.

Germination is a critical developmental stage for seed plants. For cultivation, germinated seedlings are maintained in nursery conditions during their initial growth stage [2]. Germination commences with the uptake of water imbibition of the dry seed, followed by embryo expansion, and finally, the embryo axis elongates and breaks through the covering layers to complete germination [7]. Moncaleano-Escandon* et al.* investigated the germination rate of* Jatropha* seeds stored for 0–12 months, which showed that the germination rate considerably decreased over time [8]. Stored nutrients in the seeds, including starch and soluble protein, also showed lower levels over time. In the present study, we examined the germination and initial growth of* J. curcas* because its viability and productivity largely depend on these processes. Transcriptome [9,10] and proteome [11,12,13] analyses during seed germination in *J. curcas* have been previously reported. However, to our knowledge, reports on the metabolic analysis of the *J. curcas* during seed germination are limited.

Several spectroscopy such as nuclear magnetic resonance (NMR), infrared spectroscopy (IR), near-infrared spectroscopy (NIR) have contributed a field of metabolic analysis from the early period. Nowadays chromatography-mass spectrometry is widely used for metabolic analysis. However NMR, IR, and NIR are still attractive analytical platform for metabolic analysis or profiling because of their high spectral reproducibility, simple sample preparation, and no derivatization. It is well-known that spectroscopy using different frequency electromagnetic wave has quite different properties and provides quite different information. Due to its high permeability near-infrared wave, NIR spectra can be recorded non-invasively and instantly. It is beneficial for quality examination of agricultural products. This property is also beneficial for quality examination of seeds of *J. curcas*. NMR provides plenty of structural information, such as larmor frequency, chemical shift, scalar coupling. In addition, its relatively long relaxation time allows various multidimensional NMRs. We can address each metabolite directly without chromatographic separation procedures, because 2D NMR techniques, such as HSQC, HMQC, and TOCSY, get rid of signal overlapping [14,15,16,17,18,19,20,21].

Stable-isotope-labeling has facilitated NMR analysis by enhancing its sensitivity and its abilities of signal assignment [14,15,17,19,20]. As another approach, isotopic analysis combined with heterogeneous stable-isotope-labeling provides unique information of metabolic activities. It is known as NMR metabolic flux analysis (MFA). In the NMR MFA, concentrations of isotopomers are estimated using splitting by spin-spin coupling between one bond ^1^H-^13^C (^1^*J_CH_*) and one bond ^13^C-^13^C (^1^*J_CC_*) in ^1^H and ^13^C NMR, respectively [21,22,23,24]. One advantage of NMR in metabolic flux analysis is the capacity to generate isotopic information in atomic resolution, thus, allowing estimations of a biosynthetic pathway based on their patterns of splitting. A multidimensional approach in NMR, such as zero-quantum-filtered (ZQF) TOCSY [25,26,27] and high resolution HSQC [28,29], has enabled researchers to conduct MFA without the need for sample purification.

In the present study, we applied multi-spectroscopic analyses, including NMR and NIR, to seeds of *J. curcas* for the evaluation of seed quality. Additionally stable-isotope labeling combined with NMR and isotope ratio MS (IR-MS) was also employed to monitor the flow of carbon and nitrogen in germinated seedlings. We applied heterogeneous stable-isotope-labeling of metabolites, in which seedlings were cultured in agar-plate containing ^13^C-glucose and ^15^N-nitrate, to distinguish their heterotrophic (consuming ^13^C-labeled substrates or storage substrates) or autotrophic metabolic activities. In addition, a method for high-resolution ^13^C-^13^C/^12^C bondmer analysis was proposed and examined using ^13^C-ditected ^1^H-^13^C-hetronuclar correlation spectroscopy (HETCOR) with ^13^C-optimized cryogenic probe.

## 2. Results and Discussion

### 2.1. Metabolic-Analysis-Based Quality Control Methods for Jatropha Seed

We conducted induction of seed germination using three varieties of *J. curcas* L. stored at two different temperatures (277 and 243 K) and cultivated in three different years (2009, 2011, and 2012). A total of seven samples were included in the study, namely, **1R12** (IP1P stored at 277 K, harvested in 2012), **2R12** (IP2P stored at 277 K, harvested in 2012), **2R11** (IP2P stored at 277 K, harvested in 2011), **2R09** (IP2P stored at 277 K, harvested in 2009), **2F12** (IP2P stored at 243 K, harvested in 2012), **3R12** (IP3P stored at 277 K, harvested in 2012), and **3F12**; (IP3P stored at 243 K, harvested in 2012). The germination rates of 2R12 and 3R12 were 0% and 5.1%, respectively, which were significantly lower than the other samples (75.0%, 66.3%, 46.2%, 79.7%, and 60.8% for 1R12, 2R09, 2R11, 2F12, and 3F12, respectively, Table 1). However, the germination rates of 2R09 and 2R11 were significantly higher, and, therefore, we could not conclude that storing seeds at 277 K was harmful for subsequent plant growth and development. Interestingly, the germination rate of 2R09 was 66.3%, which was significantly higher than expected, because this was observed at least three years after harvest. It has been previously reported that *Jatropha* seeds have a short viability period (<6 months) [8].

**Figure 1:**
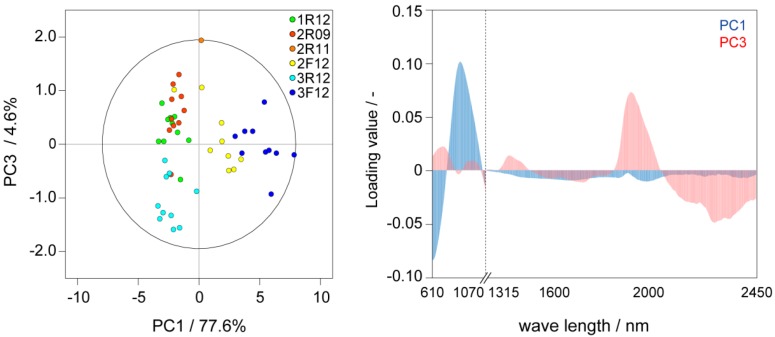
PCA of NIR spectra for the non-invasive characterization of seeds. (**a**) Score plots (PC1 *vs.* PC3) in PCA for NIR spectra (See also Figure S1). An ellipse in score plot was represented the Hotelling’s T2 95% confidence. An outlier was removed before (See Figure S2); (**b**) Loading plots (PC1 *vs.* PC3) in PCA. Input-data were generated from two different wavelength NIR spectra. Two spectra were combined after normalization. 10 seeds of 6 each different sample except for 2R12 were used for PCA.

NIR spectra provided useful information to distinguish differences in storage conditions and their varieties, although these did not provide any information on whether the seeds would undergo germination using our strategy. A score plot and a loading plot of PCA from data-matrix generated from two different wavelength NIR spectra are shown in Figure 1. The score plots were discriminated based on storage temperature (277 K or 243 K) predominantly in the principle component (PC) 1. Additionally, the score plots of IP3P seeds were weakly discriminated predominantly in PC3. The loading plot is shown in Figure 1b; however, it was difficult to identify the loading compounds due to the extensive absorbance of various molecules. Although further chemometric analyses were required to identify loading compounds, further detailed analyses were not conducted because our objective to distinguish seeds in terms of capacity to germinate was not achieved.

**Table 1:** Germination rates of 7 different seeds of *Jatropha curcas*.

	1R12	2R09	2R11	2R12	2F12	3R12	3F12
number of germinated seeds [-]	60	138	6	0	63	2	48
number of seeds [-]	80	208	13	30	79	39	79
germination rate [%]	75.0	66.3	46.2	0.0	79.7	5.1	60.8

**Figure 2:**
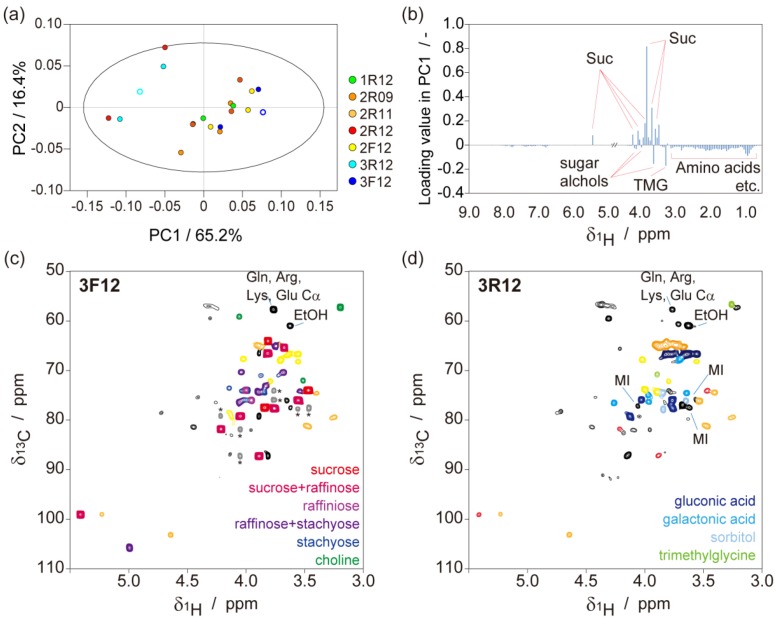
NMR analysis for water-soluble metabolites in seeds. (**a**) A score plot of PCA, in which bucket integrated (0.05 ppm/bucket) ^1^H-1D spectra were used. An ellipse in score plot was represented the Hotelling’s T2 95% confidence. The open circle plot indicates samples taken using the ^1^H-^13^C HSQC spectra of 3F12 (**c**) and 3R12 (**d**); (**b**) A loading plot of the PC1. The indicated molecules were assigned in the ^1^H-^13^C HSQC spectra. The ^1^H-^13^C HSQC spectra of 3F12 (**c**) and 3R12 (**d**). Colored signals are referenced in the lower right of the spectra. Signals indicated by asterisks in (**c**) were long-range correlations in sucrose via *^n^J_CC_* (*n* > 1). Suc; sucrose, MI; myo-inositol, TMG; trimethylglycine.

The NMR spectra of water-soluble metabolites in kernels are shown in Figure 2. The score plot in the PCA that indicated the chemotypes of 2R12 and 3R12, which showed poor viability to germinate, were discriminative Figure 2a. In the loading plot, signals from sucrose contributed to the negative direction in PC1 Figure 2b and signals from the other nutrients contributed to a positive direction. Detailed signal assignments were carried out using the ^1^H-^13^C-HSQC spectra to understand the relationship between germination rates and metabolites Figure 2c,d. In the ^1^H-^13^C-HSQC spectrum of 3F12, sucrose, raffinose, and stachyose were identified as the major sugar components. On the other hand, for 3R12, sucrose, raffinose, and stachyose were designated as trace components. However gluconic acid and galactonic acid were identified as major sugar components in 3R12. Choline was detected in 3F12, whereas this was not observed in 3R12. In contrast to choline, trimetylglycine was identified in 3R12, whereas this was not present in 3F12. Gluconic acid is a product of glucose oxidation, and trimetylglycine is a product of choline oxidation. The accumulation of gluconic acid and trimetylglycine in the present study might have been caused by oxidation over extended storage periods.

Sucrose is a major sugar form in higher-plants; it is converted to monosaccharide and then consumed as a substrate for respiration via glycolysis or used as building blocks of cell walls. Stored sucrose and glucose are utilized as the initial substrates for germination, whereas monosaccharide is derived from storage components such as starch and lipids upon commencement of germination. Raffinose family oligosaccharides (RFOs), including raffinose and stachyose, were preferentially accumulated in the seeds and are considered as important molecules for germination. RFOs are accumulated during the late stage of seed maturation and desiccation and play a role in desiccation tolerance [30,31,32,33], although several reports indicate that RFOs are not essential for germination [34].

### 2.2. NMR-Based Metabolic Analysis in Primary Growth of *J. curcas*.

**Figure 3:**
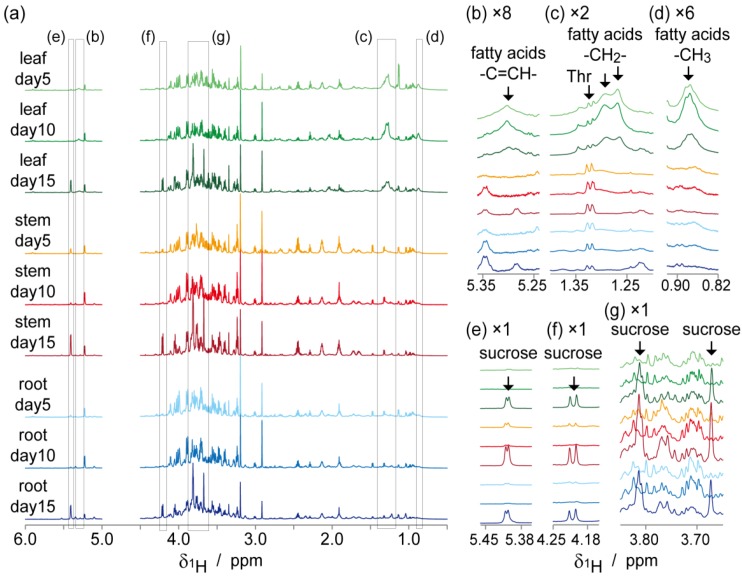
NMR analysis of water-soluble metabolites in different tissues of* Jatropha curcas* seedlings (2R09). (**a**) ^1^H-1D NMR spectra of leaves, stems, and roots harvested 5, 10, 15 days after germination. Signals from sucrose (**b**)–(**d**) were not detected or showed low levels at days 5 and 10. Signals from fatty acids (–C=CH–, –CH_2_–, and –CH_3_ for (**e**)–(**g**), respectively) were observed only in leaves.

The ^1^H-1D NMR spectra of water-soluble metabolites from roots, stems, and leaves of *J. curcas* during primary growth stages (5, 10, and 15 days after seeding) are shown in Figure 3. The signal from the H1 proton of glucose residue in sucrose (5.40 ppm) was observed in each tissue at day 15, although it was not detected in days 5 and 10. The signal from the unsaturated part of proton (–C=CH–), methylene proton, and methyl proton in fatty acid, which were observed at 5.35–5.25, 1.35–1.15, and 0.90–0.85 respectively, were strongly generated in the leaves at days 5 and 10, whereas this decreased at day 15.

These results indicate that metabolism in *J. curcas* had shifted from heterotrophic to autotrophic at a certain time point between days 10 and 15 of germination. Sucrose is the predominant product of photosynthesis and, therefore, accumulation of sucrose implies their autotrophic metabolism. On the other hand, large amounts of fatty acids in leaves were indicative of heterotrophic metabolism because gluconeogenesis from fatty acids via β-oxidation and glyoxylate cycle is a pivotal metabolic process of the seedlings. Glyoxysomes located in etiolated cotyledons contain enzymes of the fatty-acid β-oxidation cycle and the glyoxylate cycle [35]. Proteomics of germinating and post-germinating *J. curcas* have indicated that β-oxidation, glyoxylate cycle, glycolysis, citric acid cycle, gluconeogenesis, and the pentose phosphate pathway are involved in oil mobilization in seeds [11].

^13^C and ^15^N enrichments of the whole leaves, stems, and roots are shown in Table S1 and Figure S3. ^13^C enrichment in the roots was higher than that of the leaves and stems, which was 28.6% at day 15. ^13^C enrichments in the leaves and stems were limited; it was only 4.6% and 7.5% at day 15, respectively. This indicates that there are plenty of ^12^C, and not ^13^C-glucose. Contrary to this finding considerable ^13^C enrichments of glucose for NMR analysis were obtained in *Arabidopsis thaliana* [28,29,36,37]. It is considered that ^13^C and ^15^N-enrichemnts in this labeling strategy are depended on the mass of storage substrate in seeds because ^13^C and ^15^N-enrichemnts of them are natural abundant.

**Figure 4:**
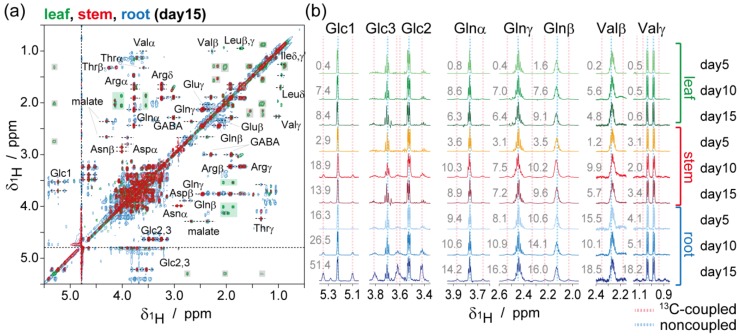
ZQF-TOCSY spectra for isotopic ratio estimation of each carbon in metabolites. (**a**) ZQF-TOCSY spectra of the roots (blue), leaves (green), and stems (red) at day 15; (**b**) The pseudo-1D ^1^H spectra generated from the ZQF-TOCSY spectra. Estimated ^13^C-enrichments are shown next to each pseudo-1D ^1^H spectra excepting Glc2 and 3. ^1^H signals coupled with ^13^C gives doublet due to scalar coupling. Therefore ^13^C-enrichments in each carbon atom in each metabolite were estimated from the ratio of integrations in ^13^C-coupled to non-coupled signals (Figure S4).

^13^C enrichments of each carbon atom in each metabolite were estimated using the ZQF-TOCSY spectra (Figure 4). In the ^1^H NMR spectra, ^1^H signals coupled with ^13^C gives doublet due to scalar coupling. Therefore, ^13^C-enrichments in each carbon atom in each metabolite was estimated from the ratio of integrations in ^13^C-coupled to non-coupled signals, although the IR-MS showed a ^13^C (and ^15^N) enrichment of total samples (Figure S3, these values were averaged ^13^C-enrichments from various metabolite and insoluble macromolecules such as proteins, nucleic acids, lignocelluloses, and plasma membranes). As described by Massou* et al.* [26,27], ZQF-TOCSY experiments are powerful methods for ^13^C-isotopic analysis that avoid significant signal overlapping of the ^1^H NMR spectra of the metabolite complex, thus enabling the estimation of ^13^C-enrichments in each carbon atom of every metabolite. ZQF-TOCSY experiments also provided better line shapes of signals than those of conventional TOCSY, thus, eliminating interference from zero-quantum coherence.

^13^C-enrichments estimated using the pseudo-1D ^1^H spectra are shown next to each spectrum in Figure 4b. Estimated ^13^C-enrichments of glucose C1 in root at 5, 10, and 15 days after seeding were 16.3%, 26.5%, and 51.4%, respectively. Additionally, estimated ^13^C-enrichments of glucose C1 in stem at 5, 10, and 15 days after seeding were 2.9%, 18.9%, and 13.9%, respectively. And estimated ^13^C-enrichments of glucose C1 in leaf at 5, 10, and 15 days after seeding were 0.4%, 7.4%, and 8.4%, respectively. This trend is the same as total ^13^C-enrichments measured with IR-MS, indicating that most glucose assimilated by the root was catabolized.

**Figure 5:**
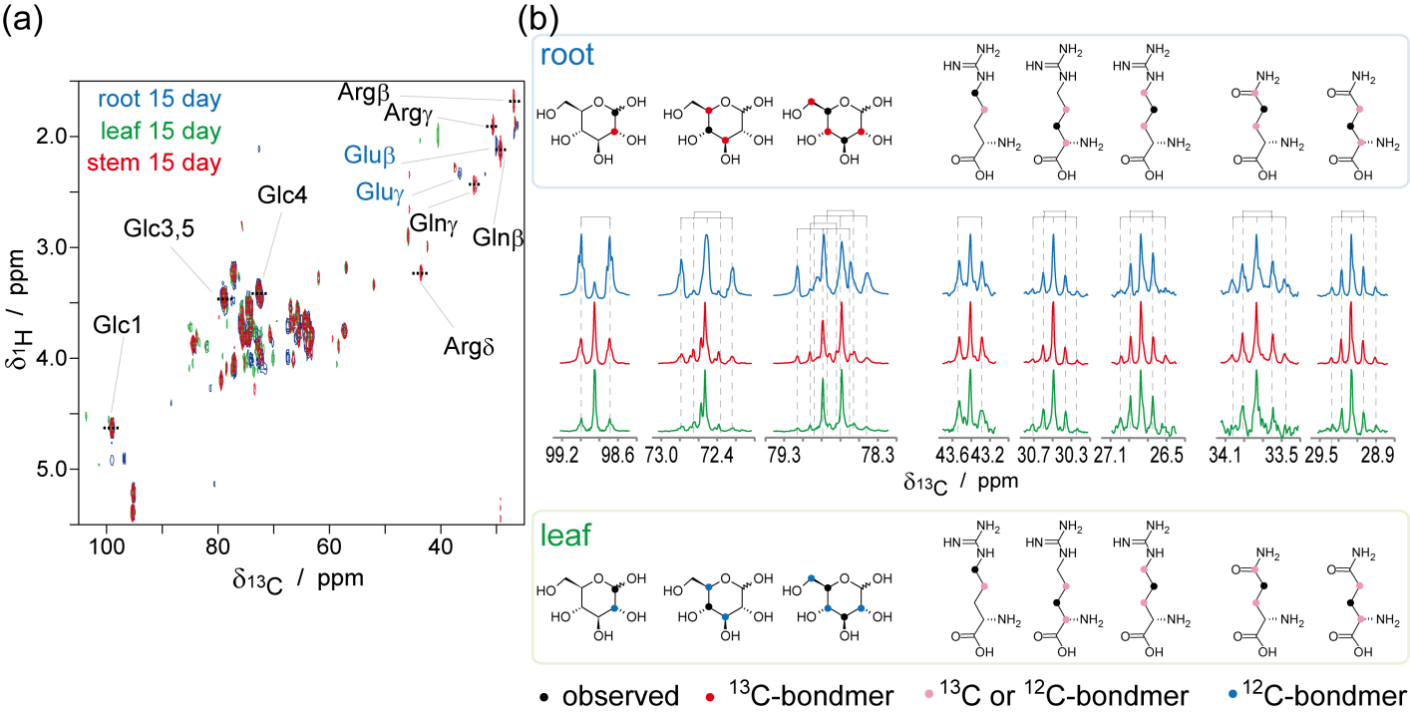
^13^C-detected ^1^H-^13^C-HETCOR spectra during ^13^C-^13/12^C bondmer analysis. (**a**) ^13^C-detected ^1^H-^13^C-HETCOR spectra of the roots (blue), leaves (green), and stems (red) at day 15; (**b**) The pseudo-1D ^13^C spectrum generated from the ^1^H-^13^C-HETCOR spectra. Generated points were indicated in (**a**) as a dotted line. Due to ^13^C-^13^C scalar couplings, the ^13^C signal is influenced by the labeling state of the adjacent carbons (Figure S4). Major bondmers estimated from signal splitting in the roots and leaves are shown as colored dots in molecular formula.

^13^C-detected ^1^H-^13^C HETCOR spectra of the leaves, stems, and roots are shown in Figure 5. The pseudo-1D ^13^C spectra of glucose, arginine, and glutamine generated from the ^1^H-^13^C-HETCOR spectra are shown in Figure 5b. In the roots, ^13^C-^13^C bond splitting were observed in all signals. In glucose, fully-labeled bondomers were predominant (Figure S4, doublets in C1 and double-doublets in C3, 4, and 5). On the other hand, in the leaves, the ^13^C-^13^C bond splitting of glucose significantly deceased. In arginine and glutamine, singlets, doublets, and double-doublets were observed, with the doublets occurring as a major component. Interestingly, the ^13^C-^13^C bond splitting patterns of arginine and glutamine in the leaves were identical to those in the roots. This indicates that arginine and glutamine were synthesized in the roots and were transferred to the leaves because there was only 4.6% of ^13^C in the leaves and trace amounts of the other amino acids in the ^13^C NMR spectrum.

^1^H-^13^C HETCOR is a powerful tool for ^13^C-^13/12^C bondmer analysis compared to conventional methods. Signal splitting from ^1^*J_CC_* in 1D-^13^C NMR were conventionally used for ^13^C-^13/12^C bondmer analyses for the studies on metabolic flux and pathway investigations [22,38]. The ^1^H-^13^C-HSQC spectrum was also used instead of 1D-^13^C spectrum to avoid signal overlap of crowded molecules [23,28,29,39,40]. It is essential to enhance the spectral resolution of the indirect dimension (^13^C) to resolve splitting from ^1^*J_CC_* (typical value is 30–60 Hz). The experimental time was also extended based on the number of increments in the indirect dimension, which was gained to enhance the spectral resolution. In a ^13^C-detected ^1^H-^13^C HETCOR experiment, the resolution of the direct dimension ^13^C was gained by increasing the acquisition time. In the present study, the resolution of the direct dimension (^13^C) was 2.99 Hz, which was enough to distinguish splitting from ^1^*J_CC_*.

^13^C-optimized (a ^13^C radio frequency coil was located inside a ^1^H radio frequency coil) cryogenic probe promoted our strategy. ^13^C-NMR is lower sensitive than ^1^H-NMR (relative sensitivity to ^1^H-NMR is 0.016) because of their low natural abundance (~1.1%) and low gyromagnetic ratio of ^13^C nuclei (~25% of ^1^H). In the cryogenic probe technology, probe cooling reduces the contribution of electronic and thermal noise and provides an increase in signal-to-noise ratio. The ^1^H-optimized cryogenic probe has been used widely for ^1^H-NMR and ^1^H-^13^C-HSQC based metabolomics as well as protein NMR. In a few studies, ^13^C-detected-NMR was applied to metabolomics for example using ^13^C-^13^C-TOCSY for carbon backbone topology analysis of metabolites [15,41]. Keun* et al.* reported ^13^C-NMR metabolomics of natural abundant urine with ^13^C-optimized cryogenic probe [42]. ^13^C-optimized cryogenic probe enabled them recorded ^13^C-1D NMR spectra on a time scale that allows its routine use. In the present study, ^1^H-^13^C HETCOR spectra were recorded with ^13^C-optimized cryogenic probe. In ^13^C-detected 2D NMR including ^1^H-^13^C HETCOR, sensitivity improvement from ^13^C-optimized cryogenic probe is effective, because number of scan were limited compared to ^13^C-1D NMR.

Nitrates assimilated by the roots are immediately reduced and converted into an organic form such as amino acids, transported through the xylem to the leaves for reduction and synthesis of amino acids, or stored in the roots as vacuoles [43]. ^15^N enrichments obtained from IR-MS measurements indicated that most nitrogen from ^15^N-nitrates remained in the roots either in the inorganic or organic form (Table S1 and Figure S3), although ^14^N was introduced from degraded amino acids from stored proteins in the seeds. Ammonium, which is the reduced product of nitrates, is fixed into glutamine, with glutamate catalyzed by glutamine synthetase (GS). Subsequently, the ammonium molecule in glutamine is fixed into glutamate with 2-oxoglutarate and catalyzed by glutamine oxoglutarate aminotransferase (GOGAT). Glutamate was observed in the roots during ^1^H-^13^C HSQC (Figure S5), as well as ZQF-TOCSY (Figure 4), however trace amounts of glutamate were observed in the leaves and stems. These findings indicate that nitrogen fixation during the GS/GOGAT cycle and glutamate assimilation occurs in the roots during this condition. Two types of GS isoenzymes exist apparently non-redundantly in plants: cytosolic (GS1) or plastidic (GS2) [44,45]. GS1 plays important roles in the primary nitrogen assimilation in the roots [45].

Glutamine and arginine, as well as asparagine, are considered the major amino acids of the xylem, playing essentials roles in nitrogen transport [46,47,48]. Furthermore, arginine serves as a major storage form of nitrogen; most seeds contain 10%–40% of their nitrogen as arginine [49]. Glutamine and arginine are estimated as major organic nitrogen forms in nitrogen transport from observed ^13^C-^13^C splitting pattern in ^13^C-detected ^1^H-^13^C HETCOR spectra. Most of the stable isotope-labeled molecules assimilated by the plants are immediately metabolized in the roots. Part of the glutamine and arginine molecules in the roots was transferred to the leaves via the stems.

Further spectroscopic analyses could enable to monitor metabolic phenomena more dynamic in germinating seeds. We previously reported* in vivo* NMR techniques to mentor storage protein degradations in ^15^N-labled germinating seeds of *Arabidopsis* *thaliana* [37]. In the previous study,* in vivo* ^1^H-^15^N-HSQC detected glutamine, asparagine, glycine, arginine, and peptides as degradative product of storage protein. A magnetic resonance imaging (MRI) technique is also applicable to monitor water dynamics in germinating seeds. We previously demonstrated modulation of water dynamics with the circadian clock in a seedling of *Arabidopsis* *thaliana* by ^1^H-NMR microscopic imaging [50]. Recently ^13^C-NMR imaging (functional imaging) was also applied plant tissue fed ^13^C-labeled substrates [51,52,53]. Development and application of new spectroscopic techniques will contribute to plant science, as well as environmental science.

## 3. Experimental Section

### 3.1. Chemicals and Plant Materials

[^13^C_6_] glucose (99% ^13^C) was purchased from Sigma Aldrich JAPAN (Tokyo, JAPAN). Deuterium oxide (99% D) and potassium nitrate (99% ^15^N) were purchased from Cambridge Isotope Laboratories (MA, USA).

Seeds from 3 different breed varieties of *J. curcas (*IP1P, IP2P, and IP3P) were used. These were stored for 1–4 years in a refrigerator or a deep freezer at 277 and 243 K, respectively. These were then subjected to NIR and NMR analysis as described later. The seeds were germinated in a 0.8 wt % agar plate without any nutrient. Germination rates were calculated by numbers of germinated seedlings and total seeds. Germinated seedlings of 2R09 were transferred 3 days after seeding on a 0.8 wt % agar plate, according to Hirayama and Kikuchi [36], containing 37.6 mM [^13^C] glucose (99% ^13^C), 0.25 mM K^15^NO_3_, 0.5 mM potassium phosphate, 0.2 mM MgSO_4_, 0.2 mM CaCl_2_, and 5 μM Fe-EDTA at 313 K. 3 seedlings were harvested 5, 10, and 15 days after seeding. Seedlings were divided into leaves, stems, and roots, and subsequently lyophilized. The lyophilized tissue was ground to powder and submitted for IR-MS and NMR analysis.

### 3.2. Spectroscopic Analysis

The NIR spectra of seeds were non-invasively recorded using a NIRSCAN-MKII (Systems Engineering, Tokyo, Japan) and FQA NIRGUN (Shibuya Seiki, Shizuoka, Japan). The wavelength ranges employed were 1250–2500 and 600–1100 nm for NIRSCAN-MKII and FQA NIRGUN, respectively. Six samples (excepting 2R12) were used for NIR analysis.

Procedures of NMR sample preparation for metabolic analysis are described below. Seeds were divided into seed coat and kernel, comprising endosperm and embryo, and then the kernels were ground to pellets. Three pellets were suspended in 1 mL of hexane. The mixture was heated at 323 K for 5 min. The supernatants were removed after the mixture was centrifuged at 15,000 rpm for 5 min. This procedure was repeated three times to remove non-polar molecules. Remaining hexane was removed using a centrifugal evaporator (TOKYO RIKAKIKAI, Tokyo, Japan). The resultant powder was suspended in 600 μL of D_2_O/KPi buffer (100 mM, pH 7.0). The mixture was heated to 323 K for 5 min and centrifuged at 15,000 rpm for 5 min. The supernatant was directly used for solution NMR experiments. Seedling powders (15 mg) were also resuspended in 600 μL of D_2_O/ KPi buffer (100 mM, pH 7.0). The mixture was heated at 323 K for 5 min and centrifuged at 15,000 rpm for 5 min. The supernatant was directly used for solution NMR experiments. Due to the limitations of the sample amount, only one NMR sample was prepared to NMR analysis.

Sample solutions were transferred onto 5-mm NMR tubes. NMR spectra were recorded on an AvanceII-700 spectrometer (Bruker, MA, USA) equipped with an inverse triple resonance CryoProbe with a Z-axis gradient for 5-mm sample diameters operating at 700.15 MHz ^1^H frequency (for ^1^H-detect experiments) or an AvanceIII-600 spectrometer equipped with an ^13^C-optimized double resonance CryoProbe with a Z-axis gradient for 5-mm sample diameters operating at 600.13 MHz ^1^H frequency (for ^13^C-detect experiments). The temperature of the NMR samples was maintained at 298 K. ^1^H-1D spectra were recorded at pre-saturation or WATERGATE methods [54] to suppress water signals. The 2D ^1^H-^13^C HSQC spectra were measured using adiabatic refocus and inversion pulses. A total of 512 complex f1 (^13^C) and 1,024 complex f2 (^1^H) points were recorded with 16 and 8 scans per f1 increment for seeds and ^13^C-labled plant tissues, respectively. The spectral widths of the f1 and f2 dimensions for the ^1^H-^13^C HSQC spectra were 175 and 16 ppm, respectively. The ZQF-TOCSY were measured according to Thrippleton and Keeler [25]. The procedure was slightly modified to measure ^13^C enrichment by introducing a ^13^C refocusing pulse during t1 evolution to remove heteronuclear scalar coupling in the indirect dimension as described by Massou* et al.* [26,27] and to suppress water signals by introducing a pre-saturation pulse during a recycling delay. A total of 256 complex f1 (^13^C) and 16,384 complex f2 (^1^H) points were recorded with 16 scans per f1 increment. The spectral widths of the f1 and f2 dimensions for the ZQF-TOCSY spectra were 12 and 12 ppm, respectively. The ^13^C-detected ^1^H-^13^C HETCOR was measured using the phase-sensitive mode. A total of 128 complex f1 (^1^H) and 16,384 complex f2 (^13^C) points were recorded with 40 scans per f1 increment. The spectral widths of the f1 and f2 dimensions for the ^1^H-^13^C-HETCOR spectra were 10 and 162.4 ppm, respectively.

^13^C and ^15^N enrichments of plant tissues were measured using an IR-MS spectrometer (IsoPrime100, Isoprime, CA, USA) connected with an elemental analyzer (vario Micro cube, Elementar Analysensysteme, Hanau, Germany).

### 3.3. Multivariable Analysis of NIR and NMR Spectra

PCA was performed with the R software [55]. For NIR spectra, two regions (610–1070 and 1315–2450) recorded different spectrometer were used for PCA. Baseline of each spectrum was corrected, and then each spectrum was normalized to unit variance (without bucket integration). Subsequently, 2 different wavelength spectra were combined. Therefore, variances of 2 different wavelength spectra in resultant vector (combined spectrum) were the same. PCA was performed based on covariance matrix without scaling (a table raw operation), smoothing, truncation, and alignment. The Hotelling’s T2 95% confidence ellipse was drawn in the score plot. An outlier was removed, and then PCA was performed again. The 1D ^1^H spectra of the seeds were subdivided into sequential 0.05-ppm designated regions between ^1^H chemical shifts of 0.5 and 9.0. After exclusion of water resonance, each region was integrated. Integrated data was normalized with constant sum method (converted to unit total spectral integral). PCA was performed based on covariance matrix without scaling (a table raw operation), smoothing, truncation, and alignment. The Hotelling’s T2 95% confidence ellipse was drawn in the score plot.

## 4. Conclusions

**Figure 6:**
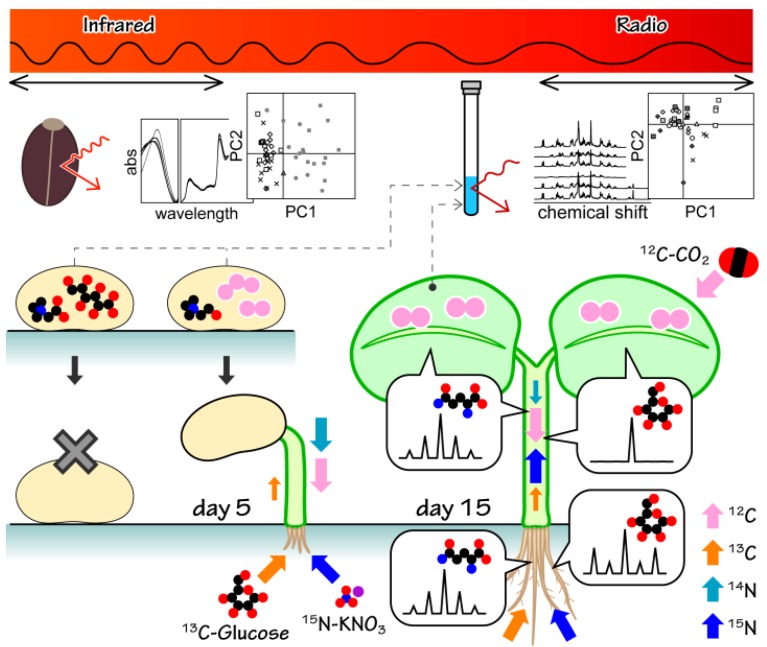
A schematic summary of the present study. Two spectroscopy using different wavelength (NIR and NMR) were applied to examine the viability of seeds of *J. curcas.* PCA score plots of NMR spectra showed oxidative reaction made seeds worsen. Stable-isotope labeling of germinated seedlings was carried out with agar-plate containing ^13^C-glucose and ^15^N-nitrate. Estimated isotope flows were indicated in arrows. NMR isotopic analysis was applied during their initial growth stage. Isotopic analysis indicated isotope flow from root to leaf was small. Isotopic pattern in ^13^C-ditected ^1^H-^13^C HETCOR indicated glutamine and arginine were the major organic compounds for nitrogen and carbon transfer from roots to leaves.

A schematic summary of the present study is shown in Figure 6. In the first half of the figure, multi-spectroscopic analysis was applied to examine the viability of seeds of *J. curcas.* It was regretful that there was no discrimination based on their germination rate in PCA score plots of NIR spectra. On the other hand, there was discrimination based on their germination rate in PCA score plots of ^1^H-1D NMR spectra. Further multidimensional NMR analysis indicated that seeds worsened due to oxidative reactions of sugars. As a result, NMR metabolic profiling determined positive and negative biomarkers of seed germination. In the second half of the figure, stable-isotope labeling-facilitated NMR metabolic analysis was applied during their initial growth stage. Nutrients in medium were labeled with ^13^C and ^15^N, however storage compound and carbon dioxide were not labeled. Therefore, metabolites were labeled heterogeneously. ^13^C enrichments measured during ^1^H-NMR, as well as IR-MS were smaller than those of previous reports involving *Arabidopsis thaliana*. This finding indicates the occurrence of strong heterotrophic metabolism during their initial growth stage, using most of the stored carbon and nitrogen. Finally, ^13^C-detected ^1^H-^13^C HETCOR was applied for ^13^C-^13^C/^12^C bondmer analysis. The ^13^C-detected ^1^H-^13^C HETCOR experiment provided high-resolution ^13^C spectra of each metabolite. It is beneficial for ^13^C-^13^C/^12^C bondmer analysis, especially combined with ^13^C-optimized cryogenic probe. NMR metabolic analysis is a powerful method for evaluating seed quality and monitoring changes in metabolism in seedlings, which could contribute to the identification of chemotypes of common breeding varieties, as well as gene-modified plants.

## Supplementary Files

Supplementary File 1
