# Supplementary material for: Multi-Spectroscopic Analysis of Seed Quality and 13C-Stable-Iotopologue Monitoring in Initial Growth Metabolism of Jatropha curcas L
Source: Metabolites. 2014 Nov 13;4(4):1018–33. doi: 10.3390/metabo4041018 (PMC4279157; doi:10.3390/metabo4041018)
Supplement: Supplementary File 1 [file metabolites-04-01018-s001.pdf]

# Supplementary Information

**Table S1.** $^{13}C$ and $^{15}N$ enrichments of various tissues of *Jatropha curcas.* $^{13}C$ and $^{15}N$ enrichments were means of three measurements of IR-MS.

| | **day** | **$\delta_{13C}$/%** | **(S.D.)** | **$\delta_{15N}$/%** | **(S.D.)** |
|---|---|---|---|---|---|
| Leaves | 5 | 1.57 | (0.009) | 3.75 | (0.066) |
| | 10 | 3.05 | (0.024) | 10.60 | (0.010) |
| | 15 | 4.64 | (0.008) | 13.26 | (0.130) |
| Stems | 5 | 3.10 | (0.016) | 8.28 | (0.027) |
| | 10 | 6.67 | (0.009) | 13.15 | (0.055) |
| | 15 | 7.52 | (0.025) | 14.49 | (0.060) |
| Roots | 5 | 15.36 | (0.124) | 19.12 | (0.142) |
| | 10 | 22.77 | (0.578) | 33.10 | (0.042) |
| | 15 | 28.57 | (0.355) | 41.45 | (0.046) |

**Table S2.** List of chemical shifts assigned to water-soluble metabolites.

| **Compounds** | **Position** | **$\delta_{1H}$/ppm** | **$\delta_{13C}$/ppm** |
|---|---|---|---|
| Glu | α | 3.75 | 56.9 |
| Gln | β | 2.44 | 33.6 |
| Gln | γ | 2.13 | 29.1 |
| Arg | α | 3.75 | 56.9 |
| Arg | β | 1.91 | 30.4 |
| Arg | γ | 1.64 | 26.5 |
| Arg | γ | 1.72 | 26.5 |
| Arg | δ | 3.24 | 43.3 |
| Thr | α | 3.58 | 63.4 |
| Thr | β | 4.24 | 68.9 |
| Thr | γ | 1.31 | 22.3 |
| Val | α | 3.58 | 63.4 |
| Val | β | 2.27 | 31.7 |
| Val | γ | 0.99 | 19.4 |
| Val | γ | 1.03 | 20.7 |
| Ala | α | 3.77 | 53.4 |
| Ala | β | 1.47 | 18.8 |
| Pro | α | 4.11 | 64.1 |
| Pro | β | 2.05 | 31.7 |
| Pro | β | 2.33 | 31.7 |
| Pro | γ | 2.00 | 26.5 |
| Pro | δ | 3.41 | 48.8 |
| Pro | δ | 3.33 | 48.8 |
| Gly | α | 3.55 | 44.3 |
| Ser | α | 3.83 | 59.2 |
| Ser | β | 3.96 | 63.1 |
| Asn | α | 3.99 | 54.0 |
| Asn | β | 2.86 | 37.2 |
| Asn | β | 2.94 | 37.2 |
| Asp | α | 3.88 | 55.0 |

**Table S2.** *Cont.*

| Compounds | Position | $\delta_{1H}$/ppm | $\delta_{13C}$/ppm |
|---|---|---|---|
| Asp | β | 2.66 | 39.1 |
| Asp | β | 2.78 | 39.1 |
| Glu | α | 3.75 | 56.9 |
| Glu | β | 2.05 | 29.8 |
| Glu | β | 2.11 | 29.8 |
| Glu | γ | 2.34 | 36.2 |
| Lys | α | 3.75 | 56.9 |
| Lys | β | 1.90 | 32.4 |
| Lys | γ | 1.44 | 24.4 |
| Lys | γ | 1.50 | 24.4 |
| Lys | δ | 1.71 | 28.9 |
| Lys | ζ | 3.03 | 41.6 |
| Leu | α | 3.75 | 56.9 |
| Leu | β | 1.69 | 42.4 |
| Leu | γ | 1.72 | 26.5 |
| Leu | δ | 0.94 | 23.6 |
| Leu | δ | 0.95 | 24.9 |
| GABA | 2 | 2.28 | 37.2 |
| GABA | 3 | 1.91 | 26.5 |
| GABA | 4 | 3.00 | 42.0 |
| Choline | 1 | 4.05 | 58.2 |
| Choline | 2 | 3.50 | 70.2 |
| Choline | Me | 3.19 | 56.6 |
| Ethanolamine | 1 | 3.82 | 60.8 |
| Ethanolamine | 2 | 3.13 | 44.0 |
| Malate | 2 | 4.29 | 73.1 |
| Malate | 3 | 2.36 | 45.3 |
| Malate | 3 | 2.66 | 45.3 |
| Succinate | 2 | 2.39 | 36.9 |
| Lactate | 2 | 4.10 | 71.5 |
| Lactate | 3 | 1.31 | 22.3 |
| Acetate | 2 | 1.90 | 25.6 |
| Sucrose | G1 | 5.40 | 94.8 |
| Sucrose | G2 | 3.55 | 73.8 |
| Sucrose | G3 | 3.75 | 75.4 |
| Sucrose | G4 | 3.46 | 72.1 |
| Sucrose | G5 | 3.83 | 75.1 |
| Sucrose | G6 | 3.80 | 62.8 |
| Sucrose | F1 | 3.66 | 64.1 |
| Sucrose | F3 | 4.21 | 79.3 |
| Sucrose | F4 | 4.04 | 76.7 |
| Sucrose | F5 | 3.88 | 84.1 |
| Sucrose | F6 | 3.80 | 65.0 |
| Glucose | α1 | 5.23 | 94.8 |

**Table S2.** *Cont.*

| Compounds | Position | $\delta_{1H}$/ppm | $\delta_{13C}$/ppm |
|---|---|---|---|
| Glucose | α2 | 3.54 | 74.1 |
| Glucose | α3 | 3.70 | 75.4 |
| Glucose | α4 | 3.40 | 72.5 |
| Glucose | α5 | 3.82 | 74.1 |
| Glucose | β1 | 4.63 | 98.7 |
| Glucose | β2 | 3.24 | 77.0 |
| Glucose | β3 | 3.46 | 78.6 |
| Glucose | β4 | 3.40 | 72.5 |
| Glucose | β5 | 3.46 | 78.6 |
| Glucose | β6 | 3.72 | 63.4 |
| Glucose | β6 | 3.88 | 63.4 |
| Fructose | α1 | 3.55 | 66.6 |
| Fructose | α2 | 3.70 | 66.6 |
| Fructose | α3 | 4.10 | 78.3 |
| Fructose | α4 | 4.10 | 77.1 |
| Fructose | α5 | 3.81 | 83.9 |
| Fructose | α6 | 3.69 | 66.1 |
| Fructose | α6 | 4.01 | 66.1 |
| Fructose | β1 | 3.55 | 65.3 |
| Fructose | β1 | 3.65 | 65.3 |
| Fructose | β3 | 3.79 | 70.2 |
| Fructose | β4 | 3.88 | 72.5 |
| Fructose | β5 | 3.99 | 71.8 |
| Fructose | β6 | 3.66 | 65.1 |
| Fructose | β6 | 3.81 | 65.1 |
| Myo-inositol | 1 | 3.54 | 74.1 |
| Myo-inositol | 2 | 4.05 | 75.1 |
| Myo-inositol | 3 | 3.54 | 74.1 |
| Myo-inositol | 4 | 3.61 | 75.1 |
| Myo-inositol | 5 | 3.24 | 77.0 |
| Myo-inositol | 6 | 3.61 | 75.1 |

**Figure S1.** PCA sore plots and lading plots of NIR analyses. (**a**), (**b**) 3D score plots of NIR analyses. 2D score plots of NIR analyses. (**c**), (**d**) PC1-PC2, and (**e**), (**f**) PC1-PC3. Coloring of score plots were based on varieties ((**a**), (**c**) and (**e**)) and germination rate ((**b**), (**d**) and (**f**)). Loading plots of PC1, PC2, and PC3.

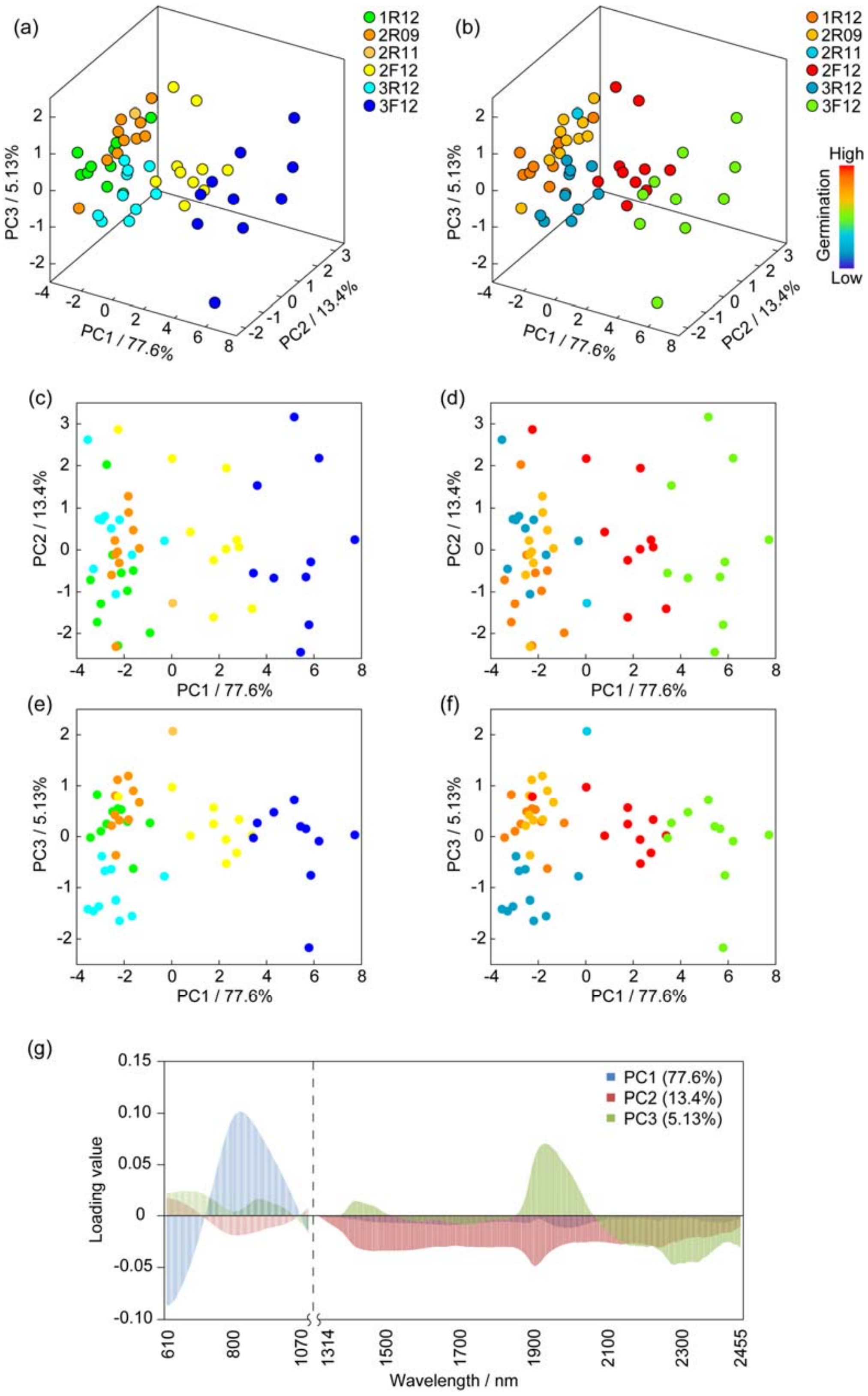

**Figure S2.** PCA sore plots (PC1-PC3) of NIR analysis (**a**) before and (**b**) after removing outlier. An ellipse in score plot was represented the Hotelling's T2 95% confidence.

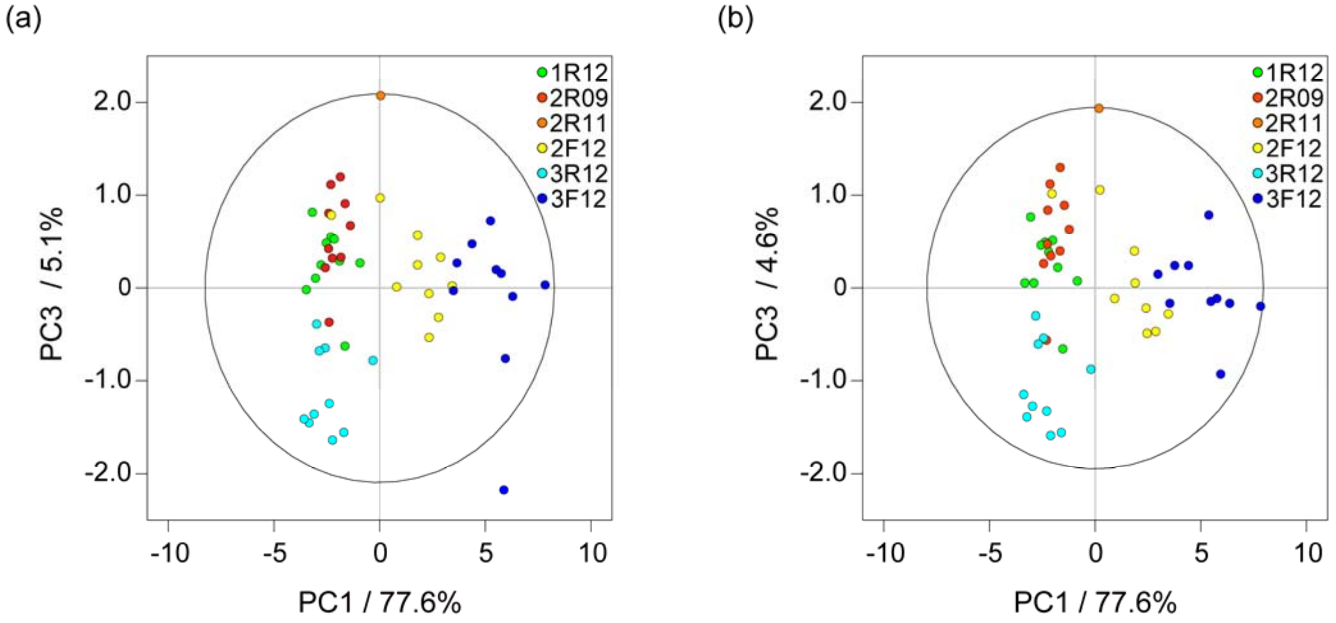


**Figure S3.** $^{13}C$ and $^{15}N$ enrichments of tissues of *Jatropha curcas* as measured by IR-MS. $^{13}C$ and $^{15}N$ enrichments were means of three measurements ± S.D.

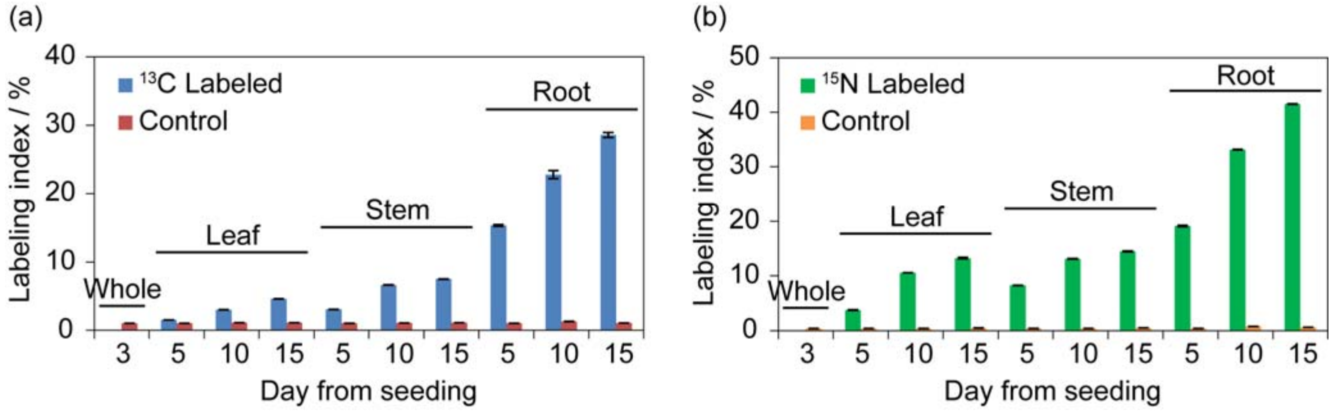

**Figure S4.** Possible splitting patterns in $^{1}H$ and $^{13}C$ NMR spectra in various $^{13}C/^{12}C$ bondomers. This figure was constructed according to Massou *et al.* [1].

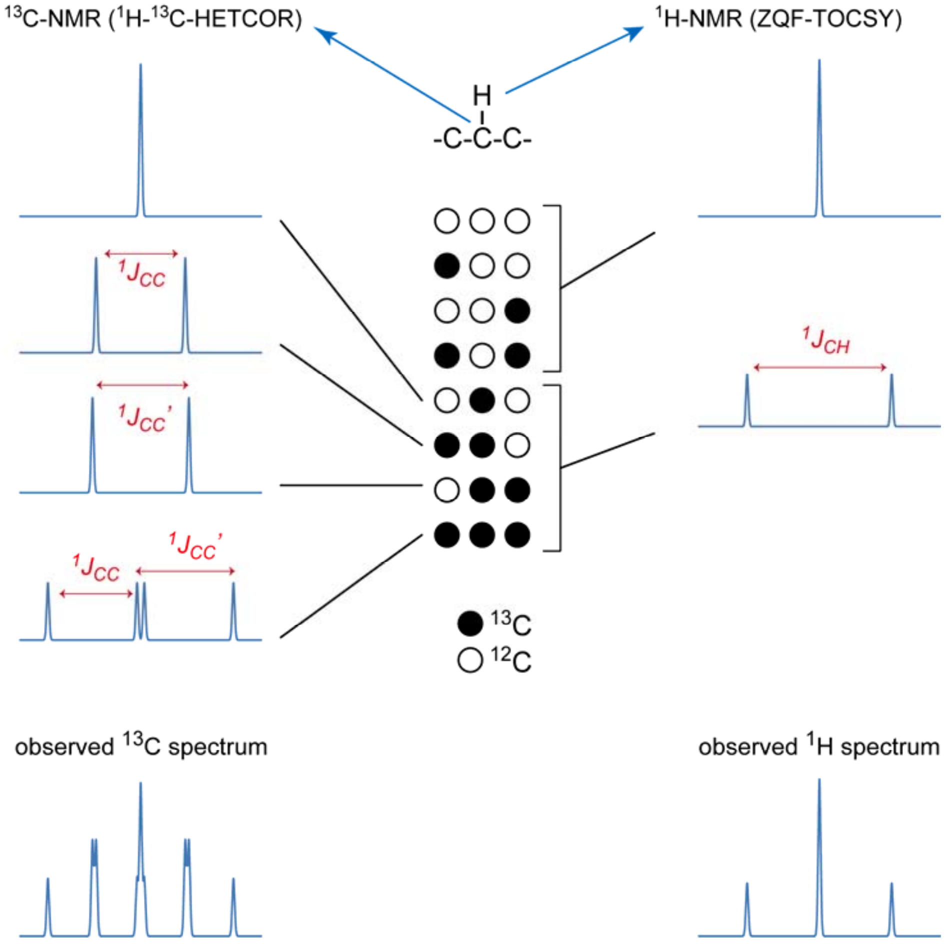


**Figure S5.** $^{1}H$-$^{13}C$ HSQC spectra of water-soluble metabolites in different tissues of *Jatropha curcas* seedlings (2R09). (**a**) Overview; (**b**) Aliphatic region ($\delta_{1H}$: 0.5–4.5 ppm, $\delta_{13C}$: 10–60 ppm); (**c**) Primary alcoholic region ($\delta_{1H}$: 3.0–4.5 ppm, $\delta_{13C}$: 60–90 ppm); (**d**) Anomeric sugar region ($\delta_{1H}$: 4.25–5.5 ppm, $\delta_{13C}$: 90–115 ppm). MI: myo-inositol; MD: maltodextrin; Suc: sucrose; Glc: glucose; Fru: fructose; EA: ethanolamine.

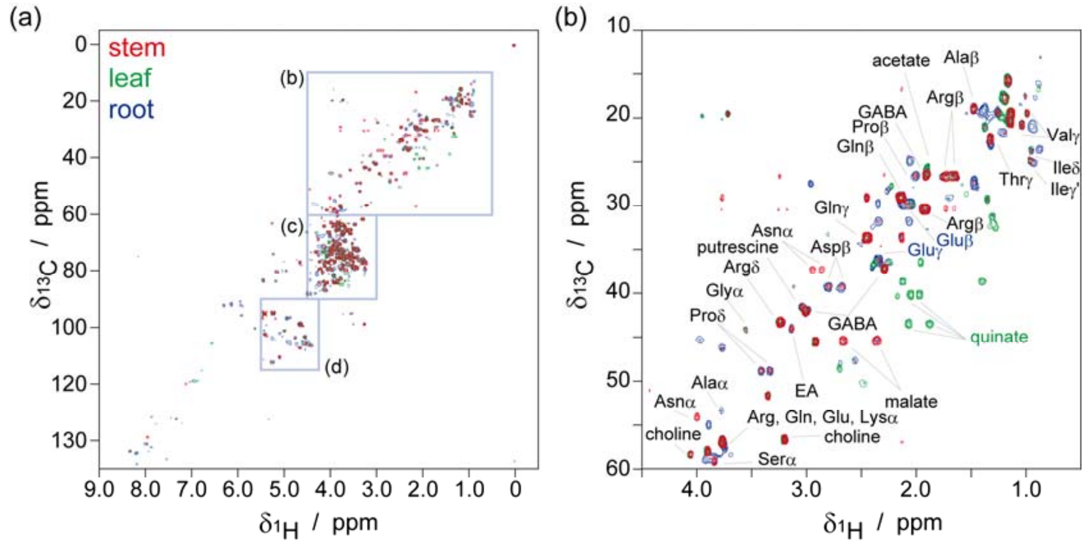

**Figure S5.** *Cont.*

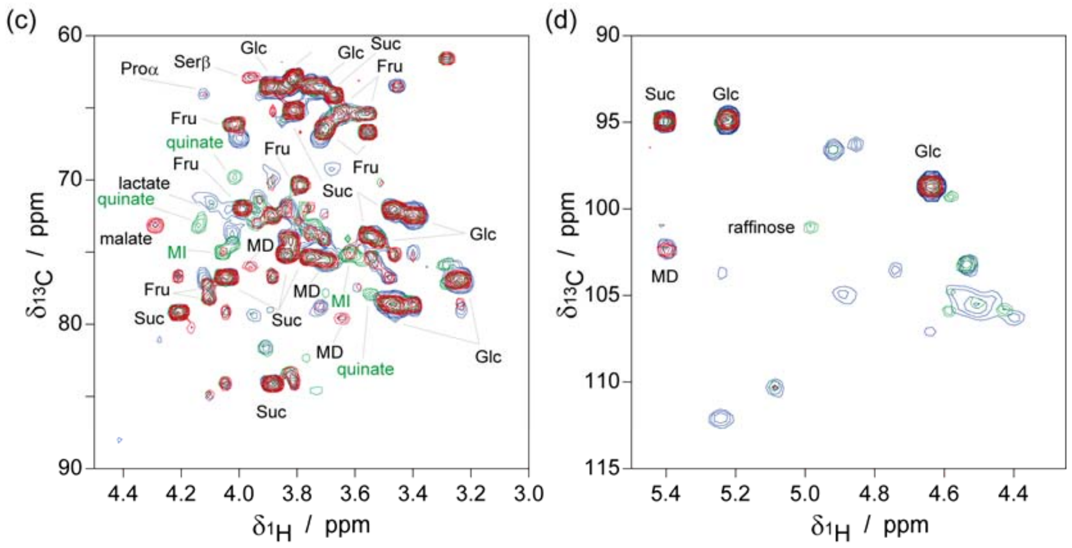


## Reference

1. Massou, S.; Nicolas, C.; Letisse, F.; Portais, J.C. NMR-based fluxomics: Quantitative 2D NMR methods for isotopomers analysis. *Phytochemistry* **2007**, *68*, 2330–2340.

© 2014 by the authors; licensee MDPI, Basel, Switzerland. This article is an open access article distributed under the terms and conditions of the Creative Commons Attribution license (http://creativecommons.org/licenses/by/4.0/).
